# Subunit protein CD40.SARS.CoV2 vaccine induces SARS-CoV-2-specific stem cell-like memory CD8^+^ T cells

**DOI:** 10.1016/j.ebiom.2024.105479

**Published:** 2024-12-11

**Authors:** Laury Nguema, Florence Picard, Marwa El Hajj, Léa Dupaty, Craig Fenwick, Sylvain Cardinaud, Aurélie Wiedemann, Giuseppe Pantaleo, Sandra Zurawski, Mireille Centlivre, Gerard Zurawski, Yves Lévy, Véronique Godot

**Affiliations:** a Vaccine Research Institute, Université Paris-Est Créteil, Faculté de Médecine, INSERM U955, Team 16, Créteil, France; b Service of Immunology and Allergy Lausanne University Hospital, Swiss Vaccine Research Institute, University Hospital, University of Lausanne, Lausanne, Switzerland; c Baylor Scott and White Research Institute, Dallas, TX, United States; d Assistance Publique-Hôpitaux de Paris, Groupe Henri-Mondor Albert-Chenevier, Service Immunologie Clinique, Créteil, France

**Keywords:** COVID-19, SARS-CoV-2, Vaccine, Long-lasting T-cell immunity, Pre-clinical models

## Abstract

**Background:**

Ideally, vaccination should induce protective long-lived humoral and cellular immunity. Current licensed COVID-19 mRNA vaccines focused on the spike (S) region induce neutralizing antibodies that rapidly wane.

**Methods:**

Herein, we show that a subunit vaccine (CD40.CoV2) targeting spike and nucleocapsid antigens to CD40-expressing cells elicits broad specific human (hu)Th1 CD4^+^ and CD8^+^ T cells in humanized mice.

**Findings:**

CD40.CoV2 vaccination selectively enriched long-lived spike- and nucleocapsid-specific CD8^+^ progenitors with stem-cell-like memory (Tscm) properties, whereas mRNA BNT162b2 induced effector memory CD8^+^ T cells. CD8^+^ Tscm cells produced IFNγ and TNF upon antigenic restimulation and showed a high proliferation rate. We demonstrate that CD40 activation is specifically required for the generation of huCD8^+^ Tscm cells.

**Interpretation:**

These results support the development of a CD40-vaccine platform capable of eliciting long-lasting T-cell immunity.

**Funding:**

This work was supported by 10.13039/501100001677Inserm, 10.13039/501100009411Université Paris-Est Créteil, and the Investissements d’Avenir program, Vaccine Research Institute (VRI), managed by the ANR.

**Research in context**

**Evidence before this study**

Since the development of effective vaccines against SARS- CoV-2 in late 2020, more than 75,900 publications are referenced in pubmed. Today, in general practice, two mRNA vaccines (Comirnaty, Spikevax) and one adjuvanted protein (Nuvaxovid) are proposed as repeated boost to maintain humoral and cellular memory and to respond to emergent variants of concern (VOCs). One unresolved question remains to understand the persistence of the vaccine-immune memory. Beyond the induction of neutralizing antibodies, there is increasing focus on the role of long-term memory T cells. It has been shown that cellular immunity is of importance for long term protection and less impacted by mutations as compare to the humoral immunity and controlling SARS-CoV-2 infection. Stem cell like memory (Tscm) CD4^+^ and CD8^+^ T cells have the ability to self-renew and have multipotency, allowing them to repopulate various subsets of memory and effector T cells. These cells are crucial in the persistence of T-cell memory. In the setting of SARS-CoV-2 infection, Tscm CD4^+^ and CD8^+^ T cells have been identified in convalescent patients and correlated with disease progression. Fourty-nine articles are referenced as vaccines (AND) Tscm in pubmed. However, the number of articles referenced as Covid vaccines and Tscm is scarce (4 publications) indicating that Tscm responses to COVID vaccines is less investigated. Furthermore, whether the mechanism of action of different vaccine platforms may influence the differentiation of Tscm and the generation of long-term protection is also unknown.

**Added value of this study**

We have previously reported that targeting SARS-CoV-2 epitopes (Spike and N antigens) to Antigen-Presenting Cells (APC) through the CD40 receptor (CD40.CoV2 vaccine) induces cross neutralizing antibodies against SARS-CoV-2, VOCs, SARS- CoV-1 and protect K18-hACE2 transgenic mice from SARS-CoV-2 challenge. We also showed the potency of this vaccine to recall cytotoxic memory T cell responses. In this new study, we report that CD40.CoV2 vaccine selectively enhances the population of long-lived spike- and nucleocapsid-specific CD8^+^ Tscm cells in two preclinical models including humanized mice. In contrast, mRNA BNT162b2 vaccine induced predominantly short-lived effector memory CD8^+^ T cells. Likely, these profiles explain differences in the durability of immune responses between APC-targeting and mRNA vaccines.

**Implications of all the available evidence**

Our findings support the development of a new generation of sub-unit polyepitopic CD40.panSarb vaccine platform, which addresses the limitations of current mRNA-based strategies by promoting the development of anti-viral long-lasting CD8^+^ Tscm cells.

## Introduction

Vaccination has played a significant role among the strategies developed to fight the SARS-CoV-2 virus for people at risk of developing a severe form of COVID-19 and in reducing viral transmission and spreading of the pandemic. However, the race against the virus continues as new variants emerge and escape the immunity induced by the first generation of vaccines. The stakes are very high, both in terms of eliminating the current virus and its variants and of preparing for the increased risk of new Coronavirus pandemics emerging in the future, given their high capacity to jump from animals to humans. The challenge lies in developing protective vaccines against the current SARS-CoV-2, its variants, and future threats from Coronaviruses and in identifying which of the portfolio of available vaccine platforms will provide long-term immunity.

An analysis of protective immune responses showed the importance of humoral immune responses, especially those involving neutralizing antibodies directed against the SARS-CoV-2 spike protein.^1^^,^^2^ The spike protein is involved in the binding and entry of the virus into human host cells through its receptor binding domain (RBD).^3^ However, some observations also suggest a significant contribution of T-cell responses in the control of SARS-CoV-2 infection.^4^, ^5^, ^6^, ^7^ Whether the humoral or cellular immune responses contribute equally to the control of SARS-CoV-2 infection or whether one is more contributive than the other is yet to be determined. The other major question concerns the sustainability of these T-cell responses. Studies of SARS and MERS infections have shown that T-cell responses are long-lasting, up to 17 years or more.^8^, ^9^, ^10^ In COVID-19, specific T-cell responses last up to eight months after infection.^11^

Among diverse memory T-cell subsets, stem cell-like memory T (Tscm) cells have been reported to have the capacity of self-renewal and the multipotency to repopulate the broad spectrum of memory and effector T-cell subsets.^12^ Their successful generation is a prerequisite for long-term protective T-cell immunity, as shown in yellow fever vaccination.^13^ In the natural history of SARS-CoV-2 infection, SARS-CoV-2 CD4^+^ and CD8^+^ Tscm cells have been detected in convalescent patients peaking four months post-symptom onset and correlate with the number of symptom-free days.^4^^,^^14^

Whether current SARS-CoV-2 vaccines induce virus-specific Tscm cells is still debated.^15^^,^^16^ Using HLA-I-multimers to detect specific CD8^+^ T cells, Jung S. et al. reported that the BNT162b2 mRNA vaccine elicits CD8^+^ T cells in infection-naïve individuals, primarily with an effector memory phenotype (approximately 80% of the specific CD8^+^ T cells), some central memory T cells, and a few cells with a Tscm phenotype.^15^ By contrast, the identification of specific T cells generated by mRNA vaccines in convalescent or infection-naïve individuals based on combined phenotypic and functional analysis showed CD4^+^ T-cell responses to be higher than CD8^+^ T-cell responses, with no induction of Tscm cells.^16^

We recently reported the effectiveness of CD40-targeting vaccination in controlling SARS-CoV-2 infection.^17^^,^^18^ This strategy uses a fully humanized monoclonal antibody directed against the CD40 receptor to target SARS-CoV-2 regions to antigen-presenting cells.^17^^,^^18^ We showed in preclinical models that the CD40.CoV2 vaccine which contains T- and B-cell epitopes from the Wuhan-spike protein (S) and nucleocapsid protein (N) highly homologous to those of 38 sarbecoviruses induced similar cross-neutralizing antibodies against variants of concern (VOCs) to that elicited by the BNT162b2 mRNA vaccine and a protection rate of 100% against virus challenge.^17^ Furthermore, the CD40.CoV2 vaccine efficiently recalled human functional and cytotoxic SARS-CoV-2 S- and N-specific CD8^+^ T-cell responses *in vitro* that were unaffected by VOC mutations and cross-reactive with SARS and, to a lesser extent, MERS epitopes.^17^ Here, we aimed to investigate the potential of this CD40.CoV2 vaccine to prime human T-cell responses *in vivo* and its ability to generate functional CD8^+^ Tscm cells relative to the BNT162b2 mRNA vaccine, using humanized mice, immunodeficient mice whose immune system has been reconstituted with a human immune system.

## Methods

### Animals and ethics

NSG HIS-mice (n = 54) were purchased from Jackson Laboratories (Bar Harbor, ME, USA). These NOD.Cg-Prkdcˢᶜⁱᵈ Il2rgᵗᵐ^1^ᵂʲˡ/SzJ mice were reconstituted at neonatal stage with human fetal liver hematopoietic stem cells from eight different HLA-A∗0201 donors. The reconstitution rates with human CD45^+^ cells of the animals were assessed by flow cytometry in the blood before immunization, reaching an average of 62%, as well as at sacrifice in the spleen (average of 70% hCD45^+^ cells; Tables S1 and S2). The frequencies of human T and B cells are detailed in Table S1 (blood) and Table S2 (spleen). Table S3 recapitulates the distribution of the HIS mice in each experiment. The CD40 humanized transgenic mice (hCD40 Tg) (n = 13) were kindly provided by Sanofi (MTA #299012).

The NSG HIS-mice and the hCD40 Tg mice were housed at the Mondor Institute of Biomedical Research infrastructure facilities (U955 INSERM-Paris East Creteil University, Ile-de-France, France). All procedures involving animals were conducted in accordance with the ethical standards and guidelines for the care and use of laboratory animals. The study protocol was reviewed and approved by the Institutional Animal Care and Use Committee (IACUC) of Anses/ENVA/UPEC (CEE-016), under approval number [20-043 #25329]. The study in the Mondor Institute of Biomedical Research infrastructure facilities was authorized by the “Research, Innovation and Education Ministry” under registration number 25329-2020051119073072 v4. All efforts were made to minimize animal suffering and to reduce the number of animals used in the experiments.

### Vaccines

The CD40.CoV2 vaccine is an anti-human CD40 12E12 monoclonal antibody (mAb) composed of a humanized variable domain (VH3/VK2) and an IgG4 constant region that we previously described.^17^ The Wuhan-vRBD domain (S AA 318 to 541) is linked to the C-terminal portion of the heavy chain constant domains. Peptides vS1 and vS2, derived from the Wuhan-spike protein (vS1: S AA 125 to 250, vS2: S AA 1056 to 1209), and peptide vN, derived from the nucleocapsid (vN AA 276 to 411), are associated with linkers and fused to the C-terminal portion of the light chain constant domains.^17^ The non-targeting IgG4.CoV2 vaccine is an IgG4 mAb linked to the same antigens in the same way as the CD40.CoV2 vaccine.

### Vaccination of mice

Mice received two injections of the CD40.CoV2 vaccine (10 μg/animal, equivalent to 1.33 μg RBD, intraperitoneal (i.p.) route) either alone or with 50 μg polyinosinic-polycytidylic acid (poly-ICLC; kind gift of Oncovir Inc, Hiltonol, i.p.), IgG4.CoV2 (10 μg/animal, equivalent to 1.33 μg RBD, i.p.), the mRNA vaccine (Pfizer/BioNtech, Comirnaty or Comirnaty XBB.1.5, 1 μg/animal, intramuscular (i.m.) route), four weeks apart. Mock animals received either PBS or poly-ICLC (50 μg/animal, i.p.). Animals were euthanized one or two weeks after the last immunization depending on the experiment. In each experiment, we performed *ex vivo* phenotyping of T- and B-cells on all the animals. The functional tests that required more cells were performed on the animals with the most splenocytes (see Table S3 for the distribution of the HIS mouse samples in each experiment).

### Anti-spike B-cell staining

Spleen cells were first incubated for 1 h at +4 °C with 2 μg/mL biotinylated pre-complexed spike tetramers (kind gift of G. Pantaleo and C. Fenwick,^19^). After washing, cells were incubated with a cocktail of fluorescent conjugated antibodies containing anti-mCD45-BV711 (clone 30-F11), anti-hCD45-PerCp-Cy5.5 (clone 2D1), anti-hCD19-BV421 (clone HIB19), anti-hCD20-APC (clone 2H7), anti-IgG-Pe.Cy7 (clone G18-145), anti-CD38-APC-H7 (clone HIT2), and anti-CD27-BV650 (clone 0323) along with PE-streptavidin (ThermoFisher Scientific). The details of the fluorochrome-conjugated monoclonal antibodies used (references, suppliers and dilutions used) are presented in Table S4.

### T-cell immunophenotyping by multi-color flow cytometry

Spleen cells were stained with fluorochrome-conjugated antibodies for specific surface markers for 30 min at RT and +4 °C. Dead cells and mouse CD45^+^ cells were excluded using LIVE/DEAD fluorescent reactive dye (Invitrogen) and an anti-mCD45-BV711. For intracellular staining experiments, cells were fixed and permeabilized using the Foxp3/Transcription Factor Staining Buffer Set (eBioscience) or BD cytofix/cytoperm™ kit (BD Biosciences), depending on the assay, and then stained for intracellular markers for 30 min at +4 °C. The following monoclonal antibodies were used for multi-color flow cytometry using HIS mouse samples: hCD45-PeCy7 (clone HI30), hCD45-APC-H7 (clone 2D1), hCD3-AF700 (clone UCHT1), hCD3-BV510 (UCHT1), hCD4-FITC (clone OKT4), hCD4-BV605 (clone RPA-TA), hCD8-PerCp.Cy5.5 (clone SK1), hCD8-APC-H7 (clone SK1), hCD45RA-PE-Dazzle-594 (clone HI100), hCD45RA-PCF (clone HI100), hCD62L-AF488 (clone DREG-56), hCD62L-BV786 (clone DREG-56), hCD95-APC (clone DX2), hOX40-BV421 (clone Ber-ACT35), hCD27-BV650 (clone 0323), hCD137-AF700 (clone 4B4-1), hIL-2-BV421 (clone MQ1-17H12), hIFNγ-PerCP-Cy5.5 (clone B27), hTNF-PeCy7 (Clone Mab 11), and hKi67-BV786 (Clone B56). The following pentamers were used: HLA-A∗02:01-KIADYNYKL (cat# FA2E, 1:10) and HLA-A∗0201-YLQPRTFLL (cat# F4339-2A-E, 1:10) PE Pentamers from ProImmune. The monoclonal antibodies used for multi-color flow cytometry using hCD40 Tg mouse samples were mCD45-BUV805 (clone 30-F11), mBB20-RB545 (clone RA3-6B2), mCD3-BV480 (clone 17A2), mCD4-BUV395 (clone RMA-5), mCD8α-BUV395 (clone 53-7.6), mCD44-BB70 (clone IM7), mCD62L-BUV615 (clone MEL-14), mCD95-APC (clone SA367H8), mSca-1-PECy7 (clone D7), mCD69-BV711 (clone H1.2F3), mCD25-BB515 (clone PC61), mOX40-BV421 (clone OX-86), mPD-1-RB780 (clone J43), mCCR7-BV605 (clone 4B12) and mTIGIT-PE (clone GIGD7). Flow cytometry was performed using an LSR II instrument with FACSDiva (BD Biosciences) and the data analyzed using FlowJo software (FlowJo LLC, version 10.8.2). The details of the fluorochrome-conjugated HLA-A∗0201 pentamers and monoclonal antibodies used in this study (references, suppliers and dilutions used) are presented in Tables S4 and S5.

### Activation-induced cell marker assay

Spleen cells were seeded at 1 × 10^6^ cells per well for 20 h in the presence of 1 μg/mL 15-mer overlapping peptide pools (OLP pools, JTP) covering the full length of the vRBD, vS1, vS2, and vN peptides included in the CD40.CoV2 vaccine, XBB.1.5 RBD or 25 ng/mL PMA plus 1 μg/mL ionomycin (Sigma Aldrich) as a positive control. An equimolar amount of DMSO was added for the negative control. After washing, cells were further stained for immunophenotyping by multi-color flow cytometry.

### SARS-CoV-2-specific T-cell functional assays

Cellular responses after the immunizations were assessed using the EpiMax assay as described in Marlin et al.^18^ Briefly, mouse splenocytes were seeded with 15-mer OLP pools covering the entire sequence of viral proteins contained in the vaccine at a final concentration of 1 μg/mL. PMA/ionomycin (eBioscience™ Cell stimulation cocktail, ThermoFisher Scientific) was used as the positive control and no stimulant was added as the negative control. Cells were restimulated or not (control conditions) after eight days of culture with the same peptides and Brefeldin A (BD Biosciences) was added. The following day, the cells were immunophenotyped by multi-color flow cytometry, including for the detection of intracytoplasmic IFNγ, TNF, and IL-2 expression to assess their functionality.

### CD8^+^ Tscm cell functional assay

To evaluate the ability of memory CD8^+^ T-cell subsets to secrete cytokines, we performed an Epimax assay, as described above, in the presence of hIL-15 and using OLP pools for the vRBD and vN peptides. The culture medium was refreshed at days 2 and 5 with medium containing hIL-15 at 25 ng/mL (PeproTech) and medium without hIL-15 at day 7.

To assess the proliferative capacity of the memory CD8^+^ T-cell subsets, spleen cells were stained with carboxyfluorescein succinimidyl ester (CFSE, ThermoFisher Scientific) diluted to 1:10,000 in pre-warmed 1× PBS for 15 min at 37 °C and further cultured with hIL-15 at 25 ng/mL (PeproTech). After nine days, cells were stained for multi-color flow cytometry. We determined the proliferation index and frequency of divided memory T-cell subsets as described by Gattinoni L. et al.^20^ for the CD8^+^ Tscm cells and the EM and CM CD8^+^ T cells.

### IFN-γ ELISpot assay

The assay was performed using spleens from non-humanized B6 background mice vaccinated with the poly-ICLC adjuvanted CD40.CoV2 or Comirnaty XBB.1.5 vaccine or mock animals. IFN-γ-releasing-specific T-cells were detected in fresh mouse splenocytes using pre-coated IFN-γ ELISpot plates according to the manufacturer's instructions (Mabtech cat#3321-4APT-10). Cells of each animal were seeded at 0.25 × 10^6^ cells/well and stimulated at 37 °C for 18-hrs with 1 μg/mL of 15-mer OLPs covering the full length of the vRBD, and vN peptides included in the CD40.CoV2 vaccine, XBB.1.5 RBD or 1 μg/mL PMA/ionomycin (eBioscience™ Cell stimulation cocktail, ThermoFisher Scientific) as a positive control. An equimolar amount of DMSO was added for the negative control. The developed spots were counted using an AID ELISpot reader (Strassberg, Germany).

### Statistics

We conducted the statistical analyses of data using GraphPad Prism 8.4.3 software (GraphPad Software).

We calculated the sample size of studied groups based on our previous study which reported in NSG humanized mice and using an ICS assay the activation of T cell responses in animals vaccinated with our poly-ICLC adjuvanted CD40.SARS.CoV2.RBD vaccine (Marlin R. et al. Nat Comm 2021). In that study, the mean frequency of specific T cells was 5.96% (SD = 3.78%) for the vaccinated group and 0% (SD = 0%) for the control group. The sample size calculation for comparing the means of two independent groups was conducted using a two-tailed Student's t-test. This calculation accounted for a desired significance level of 5% and a statistical power of 80% (β = 0.20). We found that a sample size of six HIS-mice in each group is needed to detect the difference between two groups with the described distributions. Before experimentation, we carefully measured human CD45^+^ cell levels in the blood of all animals (Table S1) and allocated them to different groups to ensure comparable reconstitution levels across all experimental conditions. This stratification approach successfully created well-balanced groups. Indeed, we performed a one-way ANOVA test on the reconstitution levels between groups, which confirmed no statistically significant differences (p > 0.05), statistically validating the homogeneity of our experimental groups in terms of CD45^+^ reconstitution levels prior to treatment (Figure S1).

To evaluate the normality of the data, we utilized the Shapiro–Wilk's method. Additionally, we employed non-parametric tests for non-normal data and made corrections for multiplicity when necessary, as explained in the figure legends. For paired data, we used the Wilcoxon signed-rank test and Friedman test followed by Dunn–Bonferroni post-hoc test for multiple comparisons. For unpaired data, we used the Mann–Whitney U test with Bonferroni correction for multiple comparisons. We conducted Spearman's test to assess the statistical relationship between two variables. Based on the assumptions of the test and the information provided in the figure legends, a p value < 0.05 was considered significant.

### Role of funders

LN received a PhD grant from the Graduate School “Life and Health Sciences” (ED 402) at the Université Paris-Est-Créteil. MEH receives PhD funding from the Graduate School of Research “Life Trajectories and Health Vulnerability” (EUR-LIVE). EUR-LIVE is a project of the PIA (Programmes d'investissements d'avenir)/ANR (Agence Nationale de la Recherche) grant 18-EUR-0011. The research program was funded by the Vaccine Research Institute (VRI) via the grant ANR-10-LABX-77. The research program was also supported by the Agence Nationale de la Recherche (ANR) grant ANR-20-COV6-0004-01. The funding sources were not involved in the study design, data acquisition, data analysis, data interpretation, or writing of the manuscript.

## Results

### The CD40.CoV2 vaccine induces polyepitopic and functional SARS-CoV-2-specific Th1 responses *in vivo*

**Fig. 1:**
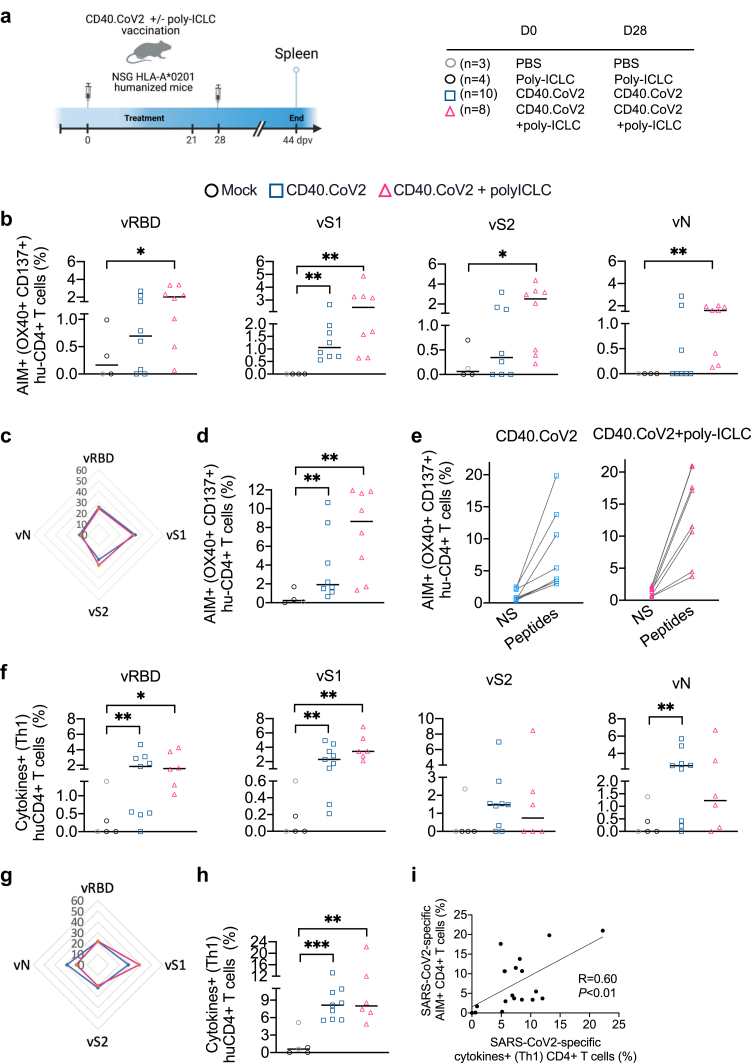
*Multiepitopic SARS-CoV-2 specific-human Th1 responses following in vivo CD40.CoV2 priming*. (a) Design of the vaccination strategy. (b) AIM assays were performed on spleen cells using OLPs covering the full-length sequences of the vaccine (v)RBD, vS1, vS2, and vN antigens. Activation of SARS-CoV-2-specific human CD4^+^ T cells is shown as the percentage of AIM^+^ (OX40^+^ CD137^+^) cells within the human CD4^+^ subset after background subtraction. (c) Radar plots showing the proportion of antigen-specific AIM^+^ CD4^+^ T cells induced by the non-adjuvanted (blue line) or poly-ICLC-adjuvanted CD40.CoV2 vaccine (pink line). (d) Percentages of AIM^+^ CD4^+^ T cells for the total reactivity. (e) AIM^+^ CD4^+^ T-cell frequencies for the total reactivity between the negative control (NS) and antigen-specific stimulations. (f**)** Percentage of human CD4^+^ T cells producing cytokines (IFNγ and/or TNF and/or IL-2) in response to SARS-CoV-2 OLPs in spleen cells of HIS-mice after background subtraction. (g) Radar plots showing the proportion of SARS-CoV2-specific cytokines^+^ (Th1) CD4^+^ T cells induced by the non-adjuvanted (blue line) or poly-ICLC-adjuvanted CD40.CoV2 vaccine (pink line). (h**)** Percentages of cytokines^+^ (Th1) CD4^+^ T cells for the total reactivity. Data were analyzed using the Mann–Whitney test. Median and individual values from two independent experiments are shown. ∗p < 0.05, ∗∗p < 0.01, ∗∗∗p < 0.001. (i) Correlation between SARS-CoV-2 specific cytokines^+^ (Th1) CD4^+^ T cells (%) and AIM^+^ CD4^+^ T cells for the total reactivity (%). Statistical comparisons were performed using Spearman's correlation.

We investigated the potency of the CD40.CoV2 vaccine to prime human cellular immune responses by immunizing mice reconstituted with a complete human HLA-A∗0201 immune system (humanized immune system (HIS)-mice) (Tables S1 and S2) with either the non-adjuvanted or poly-ICLC-adjuvanted vaccine (Fig. 1a and Table S3). We evaluated SARS-CoV-2-specific responses against the RBD (vRBD: aa 318–541) and the two other epitopes of S (vS1: aa 125–250 and vS2: aa 1056–1209) and the epitope of N (vN: aa 276–411) included in the vaccine.^17^ We selected poly-ICLC for its ability to increase the expression of CD40-and CD80/CD86-activation markers on DCs, macrophages, and B cells^18^^,^^21^^,^^22^ and enhance T-cell responses.^21^^,^^23^ Antigen-specific cells were detected by the expression of activation-induced markers (AIMs)^24^ on spleen cells stimulated with overlapping peptide (OLP) pools spanning the full-length sequence of the vaccine (v)RBD, vS1, vS2, and vN antigens. The non-adjuvanted, as well as adjuvanted, CD40.CoV2 vaccine-elicited SARS-CoV-2-specific (OX40^+^ CD137^+^) CD4^+^ T cells without antigen dominance (Fig. 1b and c). Summing the frequency of vRBD-, vS1-, vS2-, and vN-specific OX40^+^ CD137^+^ CD4^+^ T cells showed that both the non-adjuvanted and adjuvanted vaccines efficiently induced a similar frequency of SARS-CoV-2-specific human CD4^+^ T cells (Fig. 1d and e). Next, we investigated the ability of the vaccine to elicit Th1 immune responses, known to be effective against SARS-CoV-2 infection.^5^, ^6^, ^7^^,^^25^ We assessed the secretion of IFNγ, TNF, and IL-2 by CD4^+^ T cells after a nine-day re-stimulation step of spleen cells with the OLP pools. We found cytokines^+^ (IFNγ^+^ and/or TNF^+^ and/or IL-2^+^) SARS-CoV-2-specific CD4^+^ T cells to be successfully expanded after non-adjuvanted or adjuvanted CD40.CoV2 vaccination (Fig. 1f). All four OLP pools contributed to the Th1 responses without antigen dominance (Fig. 1f and g). Summing the frequency of cytokine-secreting vRBD-, vS1-, vS2-, and vN-specific CD4^+^ T cells showed the non-adjuvanted vaccine to be as effective as the adjuvanted formulation in priming Th1 responses (Fig. 1h). We confirmed a strong positive correlation between the frequency of OX40^+^ CD137^+^ cells among CD4^+^ T cells and cytokines^+^ (Th1) SARS-CoV-2-specific CD4^+^ T cells (Fig. 1i) (R = 0.60, p < 0.01, Spearman's test).

### The magnitude of RBD-specific Th1 responses correlates with spike-specific IgG-switched B cells

**Fig. 2:**
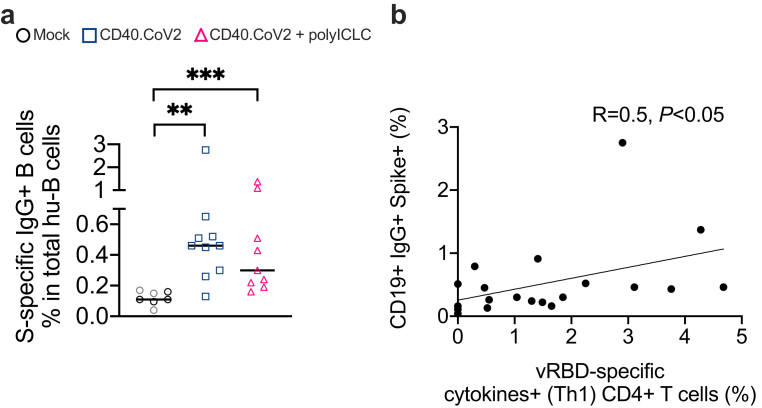
*Relationships between CD4*^*+*^*T-cell and B-cell responses to CD40.CoV2 vaccination*. (a) Percent of spike-IgG^+^ human B cells in HIS-mouse spleens. Data were analyzed using the Mann–Whitney test. Median and individual values from two independent experiments are shown. ∗∗p < 0.01, ∗∗∗p < 0.001. (b) Correlation between the percentage of vRBD-specific cytokines^+^ (Th1) CD4^+^ T cells and the percentage of spike-IgG^+^ B cells. Statistical comparisons were performed using Spearman's correlation.

We previously reported that CD40.CoV2 and BNT162b2 mRNA vaccines induced SARS-CoV-2 IgG responses with similar magnitude, cross-reactivity and neutralizing activity in the transgenic hCD40xhACE2 mice model.^17^ Here, we extend these data in the HIS mouse model, which allows better assessment of the responses against the CD40.CoV2 vaccine T-and B-cell epitopes selected for human HLA systems.^17^^,^^18^^,^^26^ A binding assay of specific IgG-switched B cells using pre-complexed spike tetramers (kind gift of G. Pantaleo and C. Fenwick,^19^), allowed us to detect a significantly higher frequency of spike-IgG^+^ switched human B cells in animals immunized with the non-adjuvanted or poly-ICLC-adjuvanted vaccine than in mock animals (CD40.CoV2 *vs* mock group p < 0.01 and CD40.CoV2+poly-ICLC *vs* mock group p < 0.001, Mann–Whitney U test) (Fig. 2a). The anti-spike B-cell responses correlated with vRBD- (R = 0.5, p < 0.05, Spearman's test) but not vS1- (R = 0.004, p = 0.98, Spearman's test) or vS2-specific cytokines^+^ (Th1) CD4^+^ T cells (R = 0.17, p = 0.47, Spearman's test) (Fig. 2b and Figure S2).

### The non-adjuvanted and poly-ICLC-adjuvanted CD40.CoV2 vaccine elicits polyepitopic CD8^+^ T cells with a different pattern of antigen-dominance

**Fig. 3:**
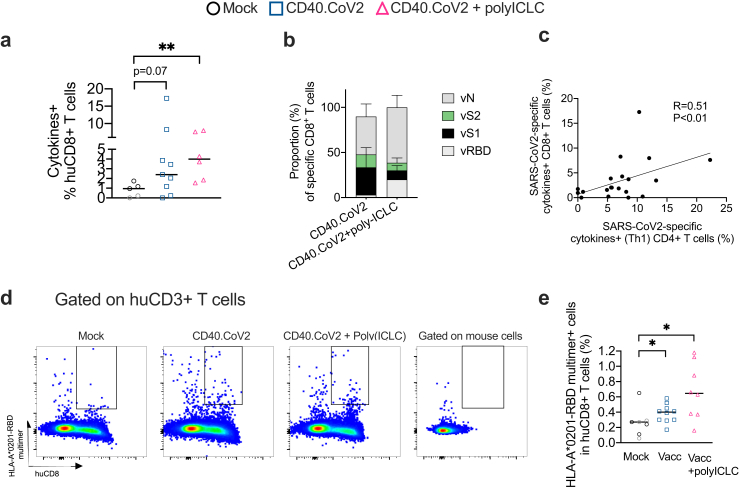
*Non-adjuvanted and poly-ICLC-adjuvanted CD40.CoV2 vaccine elicits polyepitopic CD8*^*+*^*T cells with a different pattern of antigen-dominance*. (a) Percentages of cytokines-secreting CD8^+^ T cells for the total reactivity in response to SARS-CoV-2 OLPs in spleen cells of HIS-mice after background subtraction. Data were analyzed using the Mann–Whitney test. Median and individual values from two independent experiments are shown. ∗∗p < 0.01. (b**)** Proportion of cytokines-secreting CD8^+^ T cells specific to vRBD, vS1, vS2 and vN induced by the non-adjuvanted or poly-ICLC-adjuvanted CD40.CoV2 vaccine. (c) Correlation between SARS-CoV-2 specific cytokines^+^ CD4^+^ T cells (%) and cytokines-secreting CD8^+^ T cells (%) for the total reactivity. Statistical comparisons were performed using Spearman's correlation. (d) Examples of FACS plots of HLA-A∗0201-RBD multimer staining performed on HIS-mouse spleens, gated on human CD3^+^ T cells. The negative control for the RBD multimer staining was obtained by gating on mouse CD45^+^ cells. (e) Percentages of HLA-A∗0201-RBD multimer^+^ human CD8^+^ T cells in HIS-mouse spleens. Data were analyzed using the Mann–Whitney test. Median and individual values from two independent experiments are shown. ∗p < 0.05.

We next investigated the priming of human CD8^+^ T-cell responses by the CD40.CoV2 vaccine and, more precisely, the generation of specific IFNγ- and/or TNF-secreting CD8^+^ T cells, which is also of utmost importance in controlling SARS-CoV-2 infection.^4^^,^^24^ Summing the frequency of vRBD-, vS1-, vS2-, and vN-specific human CD8^+^ T cells showed that the non-adjuvanted and adjuvanted vaccines efficiently induced cytokines-secreting CD8^+^ T cells in 67% and 100% of the vaccinees, respectively (Fig. 3a). The non-adjuvanted CD40.CoV2 vaccine steered human CD8^+^ T cell responses towards vS1-, vS2-, and vN (Fig. 3b). Conversely, the adjuvanted formulation elicited specific functional CD8^+^ T cells against the four antigens with predominant responses to vN and vRBD (Fig. 3b). We observed a positive correlation between the frequencies of SARS-CoV-2 specific cytokines^+^ CD4^+^ T cells and functional CD8^+^ T cells (R = 0.51, p < 0.01, Spearman's test) (Fig. 3c). We confirmed the presence of human CD8^+^ T cells specific for the predicted optimal epitopes from the SARS.CoV-2 RBD protein in the spleens of vaccinated HIS-mice using HLA-A∗0201-RBD multimers (Fig. 3d and e).

### The CD40.CoV2 vaccine expands central memory and Tscm CD8^+^ T cells

**Fig. 4:**
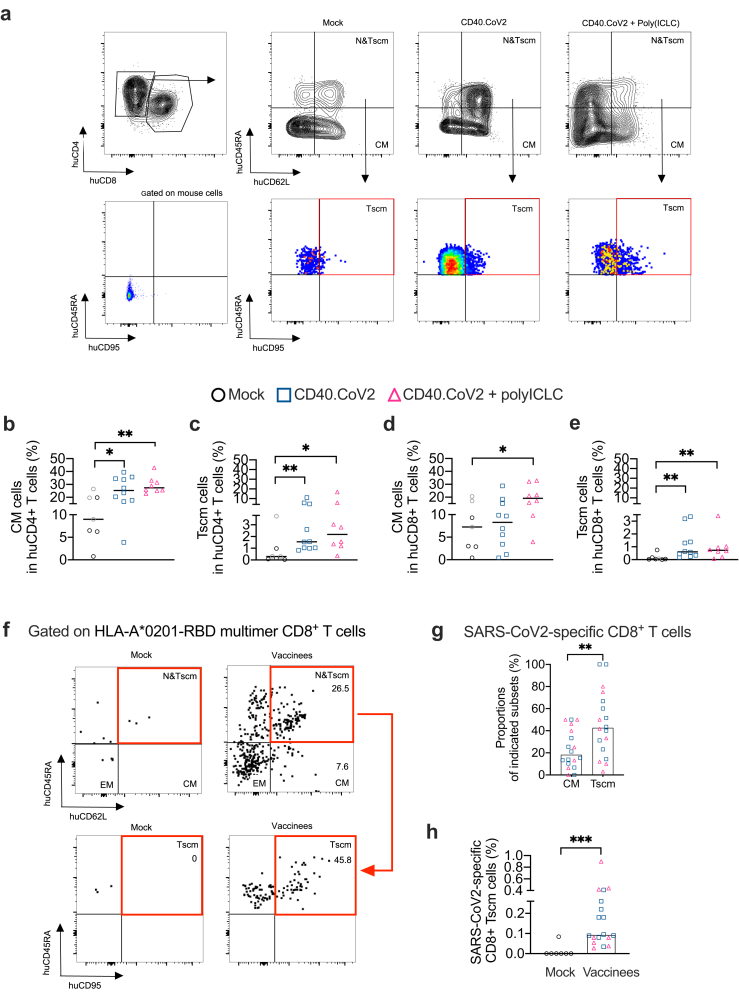
*The CD40.CoV2 vaccine induces stem-cell memory T cells*. (a) Examples of FACS plots of memory T-cell staining performed on HIS-mouse spleens, gated on human CD45^+^ cells. The negative control for the hCD95 and hCD45RA staining was obtained by gating on mouse CD45^+^ cells. (b) Percentage of CM and (c) Tscm cells among human CD4^+^ T cells. (d) Percentage of CM and (e) Tscm cells among human CD8^+^ T cells. Data were analyzed using the Mann–Whitney test. Median and individual values from two independent experiments are shown. ∗p < 0.05, ∗∗p < 0.01. (f) Examples of FACS plots of memory T-cell staining performed on HIS-mouse spleens, gated on human HLA-A∗0201-RBD multimer CD8^+^ T cells. (g) Proportion of CD8^+^ T-cell memory subsets among the human HLA-A∗0201-RBD multimer CD8^+^ T cells elicited by the CD40.CoV2 vaccine injected with (pink triangles) or without poly-ICLC (blue squares). (h) Percentage of HLA-A∗0201-RBD multimer CD8^+^ Tscm cells in HIS-mouse spleens. Data were analyzed using the Mann–Whitney test. Median and individual values from two independent experiments are shown. ∗∗p < 0.01, ∗∗∗p < 0.001.

We further evaluated the impact of the CD40.CoV2 vaccine on the different memory T-cell compartments. In this first set of experiments, we sacrificed HIS-mice 16 days after the booster injection, at a time when the contraction phase mainly affected short-lived effector memory T cells.^27^ We thus focused the analyses on long-lived memory T cells, both central memory (CM, hCD62L^+^ hCD45RA^-^) and stem-cell-like-memory (Tscm, hCD62L^+^ hCD45RA^+^, hCD95^+^) T cells (Fig. 4a). The occurrence of CM CD4^+^ T cells, as well as CD4^+^ Tscm cells, was significantly higher in the vaccinated groups than the control group (CM CD4^+^T cells: CD40.CoV2 *vs* mock group p < 0.05 and CD40.CoV2+poly-ICLC *vs* mock group p < 0.01, Mann–Whitney U test; CD4^+^ Tscm cells: CD40.CoV2 *vs* mock group p < 0.01 and CD40.CoV2+poly-ICLC *vs* mock group p < 0.05 Mann–Whitney U test) (Fig. 4b and c). The proportion of CM CD8^+^ T cells increased only in animals immunized with the vaccine plus poly-ICLC (p < 0.05, Mann–Whitney U test) (Fig. 4d), whereas the proportion of CD8^+^ Tscm cells increased in both vaccine groups (CD40.CoV2 *vs* mock group p < 0.01 and CD40.CoV2+poly-ICLC *vs* mock group p < 0.01, Mann–Whitney U test) (Fig. 4e). Next, we performed two approaches to demonstrate the capacity of CD40.CoV2 to elicit vaccine-specific long-lived memory CD8^+^ T cells. In a first approach, as described in Jung et al.,^14^ we looked at the frequency of cells exhibiting a phenotype of CM (CD45RA^−^ CD62L^+^) or Tscm (CD45RA^+^ CD62L^+^ CD95^+^) in gated HLA-RBD multimer^+^ CD8^+^ T (Fig. 4f). We found that the vaccine generated higher proportions of specific CD8^+^ T cells with a Tscm phenotype than CM, irrespective of the use of an adjuvant (p < 0.01, Mann–Whitney U test) (Fig. 4g). In a second approach, we looked at the phenotype of HLA-RBD multimer^+^ CD8^+^ T cells in total CD8^+^ T cells using CD45RA^+^, CD62L^+^ and CD95^+^ markers of Tscm. We found that the CD40.CoV2 vaccine induced a significant frequency of RBD-specific Tscm cells, with a median value of 0.093% (0.07–0.9% IQR) (p < 0.001, Mann–Whitney U test) (Fig. 4h).

### Vaccine-induced CD8^+^ Tscm cells produce cytokines and exhibit a higher proliferation potential than other CD8^+^ T memory subsets

**Fig. 5:**
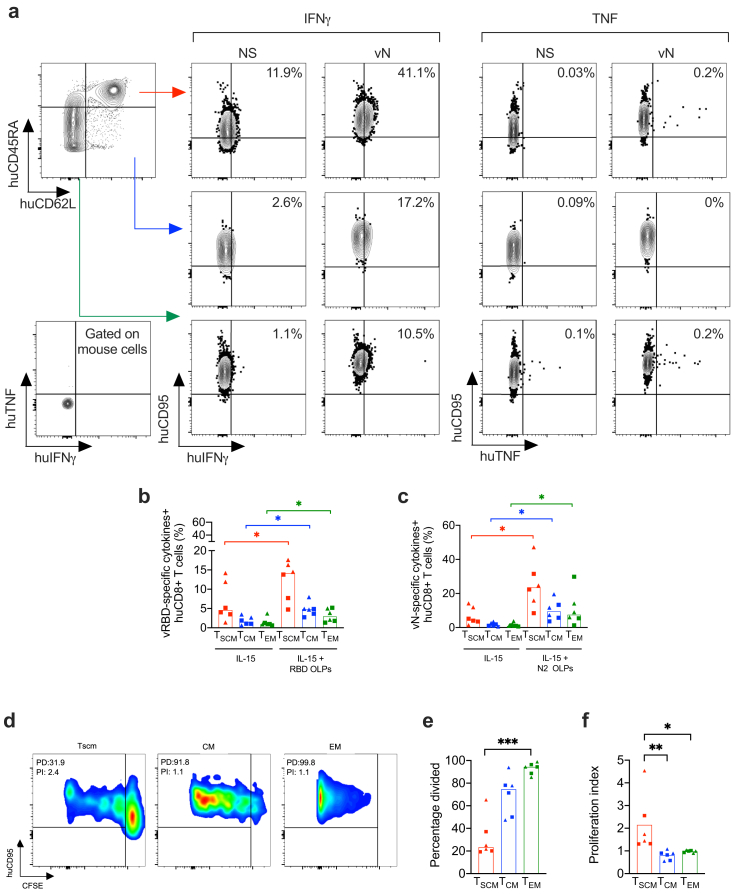
*Properties of CD8*^*+*^*Tscm cells induced by the CD40.CoV2 vaccine*. (a) Examples of FACS plots of IFNγ- and TNF-secreting memory CD8^+^ T-cell staining performed on a HIS-mouse spleen from the CD40.CoV2+poly-ICLC group in response to SARS-CoV-2 N2 OLPs, gated on human CD8^+^ T cells. The negative control for the hTNF and hIFNγ staining was obtained by gating on mouse CD45^+^ cells. (b–c) Percentage of Tscm, CM, and EM CD8^+^ T cells secreting cytokines (IFNγ and/or TNF) in response to SARS-CoV-2 (b) vRBD, or (c) vN OLPs in the CD40.CoV2 (rectangle) and CD40.CoV2+poly-ICLC (triangle) groups one week after the last vaccination. Pairwise comparisons were performed using the Wilcoxon test. Median and individual values from two independent experiments are shown. ∗p < 0.05. (d) Examples of FACS plots of CFSE dilutions in a HIS-mouse spleen from the CD40.CoV2+poly-ICLC group after stimulation with 25 ng/mL IL-15 for nine days, gated on human CD8^+^ Tscm, CM, and EM cells. PD, percentage divided; PI, proliferation index. (e) Percentage of divided cells and (f) proliferation index of different memory CD8^+^ T-cell subsets after stimulation as in panel (d). Comparisons were performed using the Friedman test followed by the post-hoc Dunn–Bonferroni test. Median and individual values from two independent experiments are shown. ∗p < 0.05, ∗∗p < 0.01, ∗∗∗p < 0.001.

We next investigated the functional properties of vaccine-induced CD8^+^ Tscm cells relative to those of EM and CM CD8^+^ T cells. CD8^+^ Tscm cells, like other memory T-cell subsets, secrete IFNγ and TNF upon antigen rechallenge^20^ and undergo numerous cell divisions in the presence of IL-15, although a large proportion of them does not divide.^20^ In this new set of experiments, we sacrificed HIS-mice vaccinated with either non-adjuvanted or poly-ICLC adjuvanted CD40.CoV2 one week after the final immunization to ensure a sufficient pool of short-lived EM CD8^+^ T cells, in addition to CM and Tscm cells. There was no significant difference in memory CD8^+^ T-cell subsets proportions between the two vaccine groups (p > 0.05, Mann–Whitney U test). Indeed, the proportion of EM CD8^+^ T cells reached an average of 26% in the vaccinees. The frequencies of CM and Tscm CD8^+^ cells reached an average of 4.8% and 22.4%, respectively. We first explored the ability of the memory CD8^+^ T-cell subsets to produce cytokines after *in-vitro* re-stimulation with their cognate antigen using the OLP pools. Two of the four antigens included in the CD40.CoV2 vaccine were selected for restimulation due to the limited number of cells available in HIS-mouse spleens. In addition to the RBD peptide against which we generated specific CD8^+^ T cells (Fig. 3, Fig. 4h), we chose the N peptide, for which the vaccine sequence is 100% homologous to that of most VOCs, including Omicron and between 80 and 100% homologous to all 38 Sarbecoviruses we tested.^17^ We found significantly higher proportions of cytokines-secreting cells in the three subsets of memory CD8^+^ T cells after re-stimulation with the vRBD and vN OLP pools than in the control condition (p < 0.05, Mann–Whitney U test) (Fig. 5a, c), regardless the use of adjuvant. We next compared the proliferative capacity of the CD8^+^ memory subsets of the vaccinees. Spleen cells from both groups of vaccinees were CFSE-labeled and cultured with IL-15 for nine days. As demonstrated by Gattinoni L. et al.,^20^ the majority of EM CD8^+^ T cells exhibited proliferation, in contrast to Tscm cells (p < 0.001, Friedman test followed by Dunn–Bonferroni post-hoc test) (Fig. 5d and e). As described, we found that a large proportion of Tscm cells did not divide. However, as expected, the proliferative index of dividing CD8^+^ Tscm cells from vaccinated HIS-mice was significantly higher than other CD8^+^ T cell populations (Tscm *vs* TCM, p < 0.01, Friedman post-hoc Dunn test; and Tscm *vs* TEM, p < 0.05, Friedman test followed by post-hoc Dunn test) (Fig. 5f). Overall, these *in vitro* assays show that the CD40.CoV2 vaccine given with or without adjuvant elicits functional specific CD8^+^ Tscm cells.

### Optimal induction of CD8^+^ Tscm cells requires targeting the CD40 receptor

**Fig. 6:**
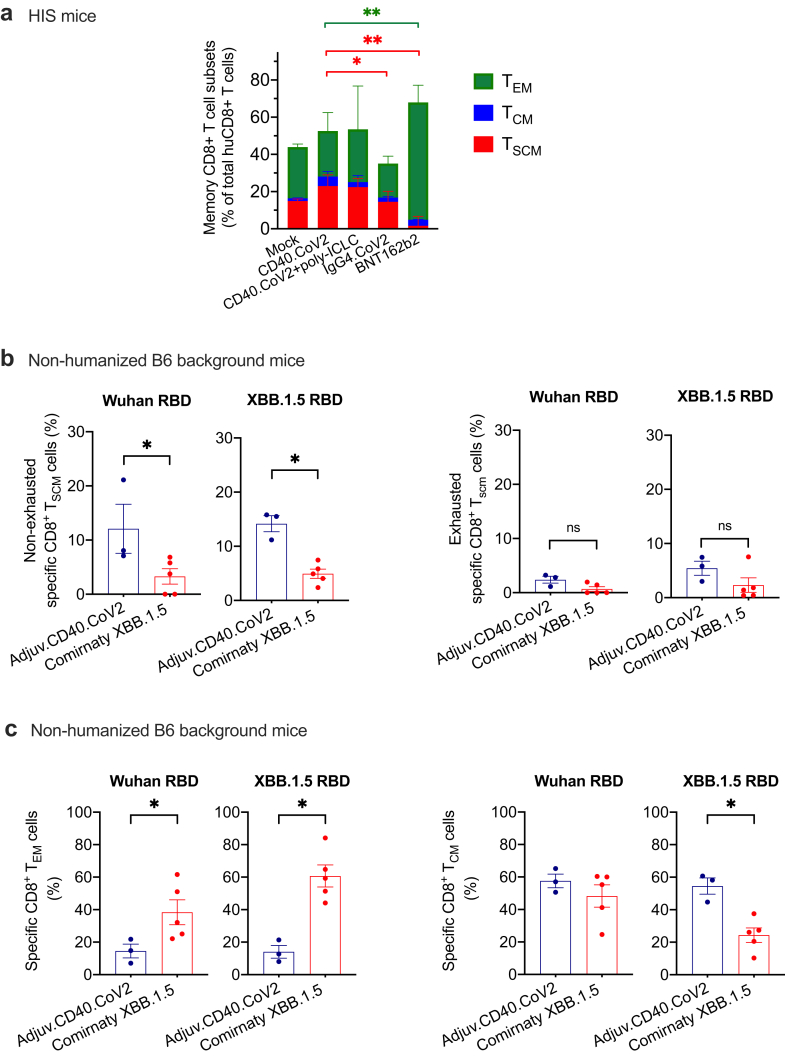
*CD40 targeting is required for optimal induction of CD8*^*+*^*Tscm cells*. (a) Proportion of CD8^+^ Tscm, CM, and EM cells among human CD8^+^ T cells in the spleens of HIS-mice one week after the last vaccination. The HIS-mice were vaccinated twice with the CD40.CoV2 vaccine alone, the CD40.CoV2 vaccine plus poly-ICLC, the Pfizer BNT162b2 mRNA vaccine, or IgG4.CoV2. Mock animals received two injections of PBS or poly-ICLC. The CD8^+^ Tscm and CD8^+^ EM cell induction was compared between the CD40.CoV2 group *versus* IgG4.CoV2 or BNT162b2 mRNA groups using the Mann–Whitney test with a Bonferroni correction. Median and individual values from two independent experiments are shown. ∗p < 0.05, ∗∗p < 0.01. (b) Proportion of Wuhan- and XBB.1.5-specific CD8^+^ Tscm cells (CD3^+^ CD8^+^ AIM^+^ CD62L^+^ CD44^-^ CCR7^+^ CD95^+^ Sca-1^+^) with a non-exhausted or exhausted (PD-1^+^ TIGIT^+^) phenotype in the spleen of hCD40 transgenic mice one week after the last vaccination (the gating strategies are shown in Figure S2b and d). The hCD40 Tg mice received an injection of CD40.CoV2 adjuvanted with the poly-ICLC (10 μg of vaccine with 50 μg of poly-ICLC) or Comirnaty XBB.1.5 mRNA vaccine (1 μg) at day 0 and day 21. Spleen cells collected at day 28 were re-stimulated with Wuhan or XBB.1.5 RBD OLPs for 20 h. The frequencies of memory CD8^+^ T cell subsets were examined by flow cytometry among the specific AIM^+^ (CD25^+^ CD69^+^) CD8^+^ T cells (see the gating strategy in Figure S2b). (c) Proportion of Wuhan- or XBB.1.5 RBD-specific CD8^+^ EM and CM T cells in the hCD40 Tg mice. The CD40.CoV2 group *versus* controls or Comirnaty mRNA group were analyzed using the Mann–Whitney test. Geometric means ± SEM and individual values from two independent experiments are shown. ∗p < 0.05.

Next, we evaluated the contribution of CD40 targeting to the induction of CD8^+^ Tscm cells by comparing CD40.CoV2 vaccination with control IgG4.CoV2 or BNT162b2 mRNA vaccination. The IgG4.CoV2 vaccine replicates the CD40.CoV2 vaccine in terms of the nature and number of antigens and their location on the heavy or light chains, except that it does not target the CD40 receptor. We noted that IgG4.CoV2 vaccination did not lead to expansion of the memory CD8^+^ T cell subsets relative to the mock group (Fig. 6a). The proportions of CD8^+^ Tscm cells were significantly higher in the CD40.CoV2 group than the IgG4.CoV2 and mRNA groups (IgG4.CoV2 *vs* CD40.CoV2 p = 0.01 and BNT162b2 mRNA *vs* CD40.CoV2 p < 0.01, Mann–Whitney U test with a Bonferroni correction) (Fig. 6a). BNT162b2 mRNA vaccination mainly activated EM CD8^+^ T cells, as previously demonstrated in humans^15^ (BNT162b2 mRNA *vs* CD40.CoV2 p < 0.01, Mann–Whitney U test with a Bonferroni correction), but not Tscm cells.

Next, we completed the evaluation of vaccine-elicited CD8^+^ T-cell responses using a second preclinical mouse model and characterized the exhausted (co-expression of TIGIT^+^ and PD-1^+^) *versus* non-exhausted phenotype of CD8^+^ Tscm cells. The huCD40 transgenic mice with a B6 genetic background but expressing human CD40, were immunized with the CD40.CoV2 vaccine or the currently available XBB.1.5 Comirnaty mRNA vaccine in head-to-head experiments. Both vaccines elicited cross-reactive T-cell responses specific to Wuhan and XBB.1.5 RBD in IFNγ-ELISpot (Figure S3a) and AIM assays (Figure S3b, c). We confirmed the superiority of the CD40.CoV2 vaccine in the induction of non-exhausted Wuhan- or XBB.1.5-specific Tscm cells (p < 0.05, Mann–Whitney U test), whereas the mRNA vaccine promoted the differentiation of specific EM CD8^+^ T cells (p < 0.05, Mann–Whitney U test) (Fig. 6b, c and Figure S3d). We have not observed any responses against the N epitope in the CD40.CoV2-vaccinated hCD40 Tg mice, in contrast to the RBD antigen (Figure S3a, c). The T-cell epitopes included in the vaccine were selected for human MHC class I and II. These data reinforce the necessity to use HIS mice to have a complete evaluation of our CD40.CoV2 vaccine.

**Fig. 7:**
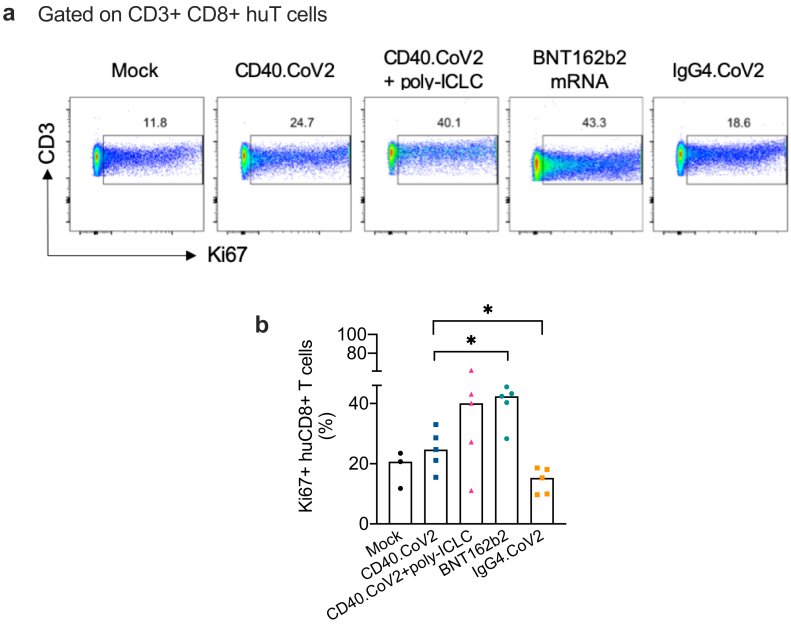
*Slower proliferation of CD8*^*+*^*T cells after CD40.CoV2 vaccination*. (a) Example of FACS plots of Ki67 staining of HIS-mouse spleens gated on human CD8^+^ T cells. (b) Percentage of Ki67^+^ CD8^+^ T cells among human CD8^+^ T cells. The CD40.CoV2 group *versus* BNT162b2 mRNA or IgG4.CoV2 groups were analyzed using the Mann–Whitney test. Median and individual values from two independent experiments are shown. ∗p < 0.05.

The induction of CD8^+^ Tscm cells requires both the initiation of signaling pathways, such as the Wnt pathway, when the antigen activates T cells and a slowing of cell proliferation after such activation.^20^^,^^28^^,^^29^ We thus evaluated the proliferation of spleen huCD8^+^ T cells using Ki67 staining in the different groups of vaccinated HIS-mice. We found less CD8^+^ T-cell proliferation in the CD40.CoV2 group than in the mRNA group (p < 0.05, Mann–Whitney U test) (Fig. 7a and b). As expected, the non-targeting IgG4.CoV2 control did not induce cell proliferation reflecting the absence of T cell activation (CD40.CoV2 *versus* IgG4.CoV2 group p < 0.05, Mann–Whitney U test) (Fig. 7a and b).

Overall, these results show that CD40-targeting vaccination provides the optimal activation of CD8^+^ T cells and drives vaccine-specific Tscm differentiation.

## Discussion

In this study, we show that a subunit vaccine targeting SARS-CoV-2 antigens through the CD40 receptor of antigen presenting cells induces the generation of functional SARS-CoV-2 specific CD8^+^ Tscm cells in two preclinical mouse models, mice reconstituted with a human immune system and the hCD40 transgenic mice. We further demonstrate the specific requirement for CD40 engagement in the successful generation of Tscm cells by a head-to-head comparison with the non-CD40-targeting vaccine IgG4.CoV2, delivering the same antigens, and mRNA Comirnaty vaccine. These results have important implications and provide insights into expanding the vaccine arsenal against coronavirus infections for effective control of the SARS-CoV-2 virus, including circulating VOCs, and the prevention of future threats. They include the development of a subunit vaccine containing non-S and conserved N sequences rich in T-cell epitopes, resulting in the capacity to induce long-term T-cell responses supported by specific CD8^+^ Tscm cells, filling the major gaps of current mRNA-based strategies.^30^^,^^31^

T cells play an essential role in protection against SARS-CoV-2 infection. Indeed, the time-lag for and paucity of T-cell responses are hallmarks of patients with severe COVID-19, whereas early induction of functional SARS-CoV-2-specific T cells is observed for patients with mild disease and rapid viral clearance.^5^, ^6^, ^7^ Sporadic cases of patients who recovered rapidly from their SARS-CoV-2 infection with no detectable antibody responses at any time, but with SARS-CoV-2 reactive T cells have also been reported.^9^ Along with evidence gathered from SARS-CoV-2 infected individuals, results from animal models allowing the depletion of CD8^+^ T cells have suggested a role for cytotoxic T cells in controlling infection with the virus.^32^ T cells activated against the ancestral SARS-CoV-2 spike protein also recognize variant spike proteins, making them even more helpful in anti-coronavirus responses.^16^^,^^33^

We have generated an all-in-one pan-Sarbecovirus CD40.CoV2 vaccine that targets T- and B-cell epitopes to the CD40 receptor of APCs.^17^ In a preclinical model, the CD40.CoV2 vaccine elicited equivalent spike neutralizing antibody responses and protection rates as the mRNA Comirnaty vaccine in a head-to-head comparison.^17^ Here, we extend the rationale for developing the CD40 platform by showing *de novo* induction of human T-cell responses in HIS-mice using multiple assays, including AIM, Epimax, and proliferation assays and staining with HLA-loaded with SARS-CoV-2 peptides. The subunit vaccine elicited human functional specific CD4^+^ and CD8^+^ T-cell responses in HIS mice without using an adjuvant. The CD40.CoV2 vaccine mainly elicited human Th1 CD4^+^ T-cell responses against the vRBD, vS1, and vN peptides, the vRBD peptide being associated with the emergence of S-IgG switched human B cells, important for conferring long-term humoral immunity.

We assessed the possible generation of long-lived memory T cells after CD40.CoV2 vaccination, with a particular interest in Tscm cells. We show that the CD40.CoV2 vaccine can elicit functional S- and N-specific CD8^+^ Tscm, which determine the durability and cross-reactivity of vaccine-induced memory CD8^+^ T-cell responses.

Tscm cells last for decades, exhibiting the capacity of self-renewal, and can give rise to effector and central memory T cells upon rechallenge with their cognate antigen. CD40-targeting vaccination, with or without adjuvant, generated CD62L^+^ CD45RA^+^ CD95^+^ CD4^+^ and CD8^+^ Tscm cells. A recent study identified two subsets of human CD8^+^ Tscm cells, a dysfunctional exhausted population, characterized by the co-expression of PD-1 and TIGIT, and a second population committed to functional lineages.^34^ We examined the expression of PD-1 and TIGIT on the CD40.CoV2-elicited CD8^+^ Tscm cells and confirmed their non-exhausted phenotype. Consistently, we showed the capacity of the CD40.CoV2-elicited CD8^+^ Tscm cells to secrete IFNγ and TNF upon restimulation with RBD- or N-OLP pools and undergo cell proliferation with a higher rate of cell division than that of effector or central memory CD8^+^ T cells. Overall, these data indicate that the SARS-CoV-2 specific CD8^+^ Tscm cells generated by the CD40.CoV2 vaccine were not exhausted cells, but rather *bona fide* stem-like memory cells.

We demonstrate that the CD40.CoV2 vaccine specifically induces Tscm. By contrast, the Comirnaty vaccines, representative of current mRNA-based vaccines, mainly activated effector memory CD8^+^ T cells and few Tscm cells. These observations are consistent with those of previous studies and the supposed mechanisms of action of these vaccines. Indeed, previous studies have suggested that CD40 activation is an optimal signal to maximize the generation of CD8^+^ Tscm cells in a tumoral context.^35^ The Comirnaty mRNA vaccine induces a strong type-1 IFN response and the secretion of CXCL10 within a few hours.^36^ Using transgenic mice deficient for innate immune sensors, Chunfeng Li et al. demonstrated the involvement of type 1 IFN in the induction of CD8^+^ T-cell responses by the Comirnaty mRNA vaccine.^36^ Type 1 IFN and CXCL10 are two signals involved in the differentiation of effector memory CD8^+^ T cells.^37^ The absence of CXCL10 or type-1 IFN, on the contrary, favors the differentiation of CD8^+^ Tscm cells.^37^ The poly-ICLC used as adjuvant in our study did not alter the ability of our vaccine to elicit functional CD8^+^ Tscm cells, consistent with the results of a previous study reporting no apparent role for the adjuvant in the differentiation of either subpopulation of memory CD8^+^ T cells.^38^

For effective control of the SARS-CoV-2 virus, including that of circulating VOCs, and to prevent future threats, it will be necessary to have an arsenal of multiple vaccine strategies, including vaccines with the capacity to maintain durable, broad-based immune responses. We demonstrate that the pan-Sarbecovirus CD40.CoV2 vaccine elicits functional S- and N-specific CD8^+^ Tscm providing further evidence in favor of the clinical evaluation of this vaccine candidate. Clinical studies testing in humans the potency of CD40.COVID vaccines as booster of pre-existing immunity, induced either by previous priming with available vaccines or natural infection, are planning in 2024. This is supported by the recent demonstration that a single dose of a first-generation CD40.RBD vaccine, injected without adjuvant, was sufficient to elicit neutralizing antibodies that protected macaques against a new viral challenge.^18^

### Limitations of the study

We have used only one HIS-mouse model to demonstrate Tscm induction by the CD40.CoV2 vaccine. However, our goal was to demonstrate differences of Tscm induction between mock and vaccinated animals in two models: NSG HIS-mice and immunocompetent mice. Our HIS mice model was reconstituted with only HLA-A∗0201 donors. Thus, our study mainly examined the specificity and functionality of CD8^+^ Tscm cells but not CD4^+^ Tscm cells. Due to an insufficient number of cells, we could not study the cytotoxic activity of the CD8^+^ T cells generated by the CD40.CoV2 vaccine in the HIS-mouse model. However, we have already demonstrated the capacity of CD40.CoV2 to recall human SARS-CoV-2 specific CD8^+^ T cells exhibiting specific cytotoxic activity *in vitro*.^17^ As HIS-mice do not express the hACE2 protein, we could not carry out viral challenge experiments. However, we have previously shown the capacity of this CD40-targeted vaccine to protect monkeys and hACE2 transgenic mice against SARS-CoV-2 challenge.^17^^,^^18^

## Contributors

Conceptualization: VG and YL.

Experimentation: LN, FP, MEH, and LD.

Formal analysis: VG, LN, FP, CF, GP, LD, and AW.

Design and production of the CD40.CoV2 vaccine: SZ, GZ, SC, and YL.

Resources: CF, GP, SC, SZ, and GZ.

Funding acquisition: VG and YL.

Project administration: MC.

Writing – original draft: VG, YL, and LN.

Access and verification of underlying data: VG, LN.

All authors have read and approved the final version of the manuscript.

## Data sharing statement

The CD40.CoV2 vaccine generated in this study was deposited in GenBank: anti-human CD40 12E12 antibody IgG4 H chain (GenBank ID: AJD85779.1 residues 20–467) fused to SARS_CoV_2RBD (GenBank ID: UEP92470.1 residues 17–240) followed by EPEA (C-tag) and the anti-human CD40 12E12 antibody kappa L chain (GenBank ID: AJD85780.1 residues 21–236) fused sequentially to a linker (GenBank ID: AJD85777.1 residues 699–725), nucleocapsid phosphoprotein, partial [Severe acute respiratory syndrome coronavirus 2] (GenBank ID: QWE63393.1 residues 95–230), linker residues AR, Chain A, Spike protein S1 [Severe acute respiratory syndrome coronavirus 2] (GenBank ID: 7M8J_A residues 113–237), linker residues TR, and Sequence 12 from patent US 8518410 (Genbank ID: AGU17682.1 residues 3–27), surface glycoprotein, partial [Severe acute respiratory syndrome coronavirus 2] (GenBank ID: UET03776.1 residues 195–348). The authors declare that other data supporting the findings of this study are available from the corresponding author upon request.

## Declaration of interests

The authors SZ, GZ, MC, SC, and YL are named inventors on patent applications based on this work held by Inserm Transfert. The remaining authors declare no competing interests. Inserm Transfert provided a license for CD40-targeting vaccines to the biotech company LinKinVax.
