# Supplementary material for: Subunit protein CD40.SARS.CoV2 vaccine induces SARS-CoV-2-specific stem cell-like memory CD8+ T cells
Source: eBioMedicine. 2024 Dec 11;111:105479. doi: 10.1016/j.ebiom.2024.105479 (PMC11697708; doi:10.1016/j.ebiom.2024.105479)
Supplement: Supplementary Tables and Figures [file mmc1.docx]

**Table S1. Reconstitution rate with human cells in HIS mouse blood before and after vaccination**

**Table S2. Reconstitution rate with human cells in HIS mouse spleens after vaccination**

**Table S3. Distribution of HIS mouse samples in the various tests**

In the various *ex vivo* tests, the distribution of animals and their participation in immune cell phenotyping and functional assays were recorded and categorized as follows: 'x' indicating the sample used for the test, and '0' indicating the sample not used for the test.

**Table S4. List of antibodies and pentamers used for human cell immunophenotyping by multi-color flow cytometry**

| *h: human*  *m: mouse* |
| --- |
| *AF: alexa fluor; APC: allophycocyanin;*  *Cy: cyanine; FITC: fluorescein isothiocyanate; PE: phycoerythrin; BV: brilliant violet; PerCP: peridinin-chlorophyll-protein* |

**Table S5. List of antibodies and pentamers used for mouse cell immunophenotyping by multi-color flow cytometry**

| *m: mouse* |
| --- |
| *APC: allophycocyanin;*  *Cy: cyanine; PE: phycoerythrin; BUV:*  *Brilliant Ultra Violet BV: brilliant violet; RB: RealBlue™; BB: Brilliant Blue.* |

**Supplemental Figure 1.**

**Fig. S1: Levels of human CD45+ reconstitution measured in the blood of all animals before experimentation (detailed values are given in Table S1) showing comparable reconstitution levels across all experimental conditions and well-balanced groups**. Data are represented as mean +/- SD. A one-way ANOVA test on the reconstitution levels between groups confirmed no statistically significant differences (p > 0.05).

**Supplemental Figure 2.**

**Fig. S2. Relationships between CD4^+^ T-cell and B-cell responses to CD40-CoV2 vaccination.** Correlation between percent of spike-IgG+ B cells and percent of **(**a) S1-, (b) S2-specific Th1 CD4^+^ T cells. Statistical comparisons were performed using Spearman correlation.

**Supplemental Figure 3.**

**Fig. S3. The CD40.CoV2 vaccine elicited a higher proportion of specific CD8^+^ Tscm cells in the hCD40 transgenic mouse model.**

The hCD40 Tg mice received an injection of CD40.CoV2 adjuvanted with the poly-ICLC (10 μg of vaccine with 50 μg of poly-ICLC) or Comirnaty XBB.1.5 mRNA vaccine (1 μg) at day 0 and day 21. Animals were sacrificed one week after the last injection. Control animals received PBS or poly-ICLC injections. (a) Specific IFNγ-T cell responses were evaluated by ELIspot assays using Wuhan-vRBD, XBB.1.5 RBD or vN OLPs. Results are expressed as number of spots per millions of cells after background deduction. (b-c) Specific CD8^+^ T cell responses were evaluated using the AIM assay. Spleen cells were re-stimulated with Wuhan or XBB.1.5 RBD OLPs, N OLPs, αCD3/αCD28 coated beads (positive control) or left unstimulated (negative control) for 20-hrs. (b) Example of gating strategies to identify the AIM^+^ specific CD8^+^ T cells and the proportion of AIM^+^-specific memory CD8^+^ T cell subsets. (c) Frequencies of AIM^+^ CD8^+^ T cells. Geometric means +/- SEM are represented. The CD40.CoV2 group *versus* controls or Comirnaty mRNA group were analyzed using the Mann-Whitney test. *p < 0.05, **p<0.01. (c) Example of gating strategies to identify the SARS-CoV2-specific CD8^+^ Tscm cells (CD3^+^ CD8^+^ AIM^+^ CD62L^+^ CD44^-^ CCR7^+^ CD95^+^ Sca-1^+^) with a non-exhausted or exhausted (TIGIT^+^ PD-1^+^) phenotype.
